# Editorial: Herbal Medicines in Managing Stroke and Neurodegenerative Diseases—Is There Evidence Based on Basic and Clinical Studies?

**DOI:** 10.3389/fphar.2021.783829

**Published:** 2021-10-28

**Authors:** Haiyu Xu, Hui Zheng, Chunguang Li, Huazheng Liang

**Affiliations:** ^1^ Institute of Chinese Materia Medica, China Academy of Chinese Medical Sciences, Beijing, China; ^2^ The Third Hospital/Acupuncture and Tuina School, Chengdu University of Traditional Chinese Medicine, Chengdu, China; ^3^ NICM Health Research Institute, Western Sydney University, Westmead, NSW, Australia; ^4^ Translational Research Institute of Brain and Brain-Like Intelligence, Shanghai Fourth People’s Hospital, School of Medicine, Tongji University, Shanghai, China

**Keywords:** herbal medicine, ethnopharmacology, plant derivatives, molecular mechanism, stroke, neurodegenerative disorders

Stroke and dementia are common diseases afflicting the elderly population. Stroke is the leading cause of death in China (Zhou et al., 2016), and Alzheimer’s disease (AD) is estimated to be the 2nd economic burden by 2020. Ischemic stroke has been successfully managed with thrombolytic agents (Hacke et al., 1995; Tsivgoulis et al., 2018) and endovascular thrombectomy (Zi et al., 2021; Suzuki et al., 2021), but a decent proportion of patients still cannot completely restore their neurological functions. These patients with remaining symptoms will be the potential target population of herbal recipes as an adjuvant therapy.

In searching for potential therapeutics that could serve as adjuvant therapies for patients suffering from these diseases, it has been revealed that some herbal recipes including Chinese herbs and other traditional medicines have been widely used by TCM practitioners and physicians in other countries (Iwasaki et al., 2004; Fu et al., 2013; Wang et al., 2015). For the same clinical manifestations, different herbal recipes can be used based on the diagnosis by individual TCM practitioners. There must be is a significant difference in the etiology and pathogenic mechanisms of these diseases. Though there is a large number of publications on clinical use of herbal recipes, few of them comply with the criteria of randomized controlled trials (Fu et al., 2013). It is, therefore, necessary to assess the scientific evidence for these herbal recipes in managing the above-mentioned illnesses using state of the art pharmacological techniques. This will not only facilitate the discovery of new therapeutics or compounds, but also refresh our knowledge in understanding how these diseases develop.

This Research Topic is a collection of nine articles, including four original articles, four review articles and one clinical trial protocol, aiming to examine the commonly used herbal recipes and the molecular mechanisms underlying their therapeutic effects, especially on neurodegenerative diseases.

Stroke is the second leading cause of death worldwide and the first in China. Though intravenous thrombolysis and endovascular thrombectomy have significantly improved the outcome of patients with acute ischemic stroke, a decent proportion of patients were ineligible for these therapies or disabled even after receiving these treatments. New therapies are needed to manage these patients. Feng et al. reported that Panax notoginseng saponins (PNS) (Xueshuantong lyophilized powder), an extract from the roots and rhizomes of Panax notoginseng, were shown to increase brain perfusion and neural plasticity through anti-inflammatory, antioxidant, and anti-apoptosis mechanisms. However, the therapeutic effect of PNS has not been confirmed in large-scaled randomized clinical trials. They aim to conduct an RCT by recruiting 480 patients with acute ischemic stroke. Their protocol was submitted to this journal. In a review, Wang et al. summarized evidence of ginseng Rb1, one of the five effective components of PNS, in managing ischemic stroke. It was found that ginseng Rb1 exhibited its protective effect through antioxidant, anti-inflammatory, anti-apoptosis capacities. In addition, it also suppressed excitotoxicity and calcium influx, maintained the integrity of the blood-brain barrier and energy metabolism. Zhang et al. explored the protective effect and the underlying mechanism of An-gong-niu-huang wan pre-treatment on cerebral ischemia and found that it mitigated ischemic injury by reversing the up-regulation of reactive oxygen species and malondialdehyde as well as increasing the expression of p-GSK-3β(Ser9)/GSK-3β (glycogen synthase kinase-3β) ratio and heme oxygenase-1. Yu et al. investigated the synergistic effect of Ligusticum chuanxiong Hort and borneol. It was found that Ligusticum chuanxiong Hort promoted neurogenesis and preservation of mature neurons, whereas borneol improved the ultrastructure of the blood brain barrier and increased expression of tight junction associated proteins, vascular endothelial growth factor, and vascular endothelial growth factor receptor 2, providing an optimal environment for neurogenesis.

AD is the leading neurodegenerative disease inflicting nearly 43.8 million people in the world. Numerous studies have investigated the mechanisms underlying AD but few drugs have been found due to the complex nature of this disease (Abeysinghe et al., 2020). Endeavoring to isolate effective compounds, Wei et al. tested whether the main active fraction combination (LW-AFC) extracted from Liuweidihuang decoction (LW) can improve cognitive and emotional functions in a cranial-irradiation mouse model. It was found that LW-AFC improved both cognitive and depressive behaviours by increasing the number of neural stem cells in the dorsal hippocampus and rectified the altered microenvironment by increasing the contents of glutathione and other factors. In a meta-analysis, Kwon and Lee reported their findings from 52 studies, including 36 RCTs, on the therapeutic effect of herbal medicine on behavioural and psychological symptoms of dementia (BPSD). Though the level of evidence is not optimal, herbal medicine does serve as a promising therapy complementing the conventional western treatments.

Parkinson’s disease is the second leading cause of neurodegenerative disease, severely impacting patients’ movement and daily life. Due to limited efficacy of currently available medication and their side-effects, many patients prefer to take herbal medicines. Lin et al. analyzed the usage of Chinese herbal products (CHPs) in the general population by reviewing the National Health Insurance Research Database of Taiwan. They found that the most commonly used formula was Chaihu-Jia-Longgu-Muli-Tang, and *Uncaria tomentosa* is the most widely used herb which has been used to treat non-motor symptoms for PD patients. In understanding the pathogenic mechanism of PD and its treatment, Wu et al. summarized indirect evidence of ferroptosis involved in PD pathogenesis and TCM recipes for managing PD. Puerarin, isolated from a plant and a natural ferroptosis inhibitor, showed potential as a new drug candidate for managing PD. This warrants further studies. Su et al. reviewed experimental evidence of resveratrol’s therapeutic mechanisms in animal models. It was found that resveratrol exerted protective effects on mitochondrial and motor functions through its anti-oxidative, anti-inflammatory, and anti-apoptosis capacities.

It is our hope that the articles included in this Research Topic provide an update on therapeutic effects, molecular mechanisms, potential active components, as well as clinical evidence and possible future directions for using herbal medicines for the management of neurodegenerative diseases.
